# Path analysis: A method to estimate altered pathways in time-varying graphs of neuroimaging data

**DOI:** 10.1162/netn_a_00247

**Published:** 2022-07-01

**Authors:** Haleh Falakshahi, Hooman Rokham, Zening Fu, Armin Iraji, Daniel H. Mathalon, Judith M. Ford, Bryon A. Mueller, Adrian Preda, Theo G. M. van Erp, Jessica A. Turner, Sergey Plis, Vince D. Calhoun

**Affiliations:** Tri-Institutional Center for Translational Research in Neuroimaging and Data Science (TReNDS), Georgia State University, Georgia Institute of Technology, and Emory University, Atlanta, GA, USA; School of Electrical and Computer Engineering, Georgia Institute of Technology, Atlanta, GA, USA; Department of Psychiatry, University of California, San Francisco, CA, USA; San Francisco VA Medical Center, San Francisco, CA, USA; Department of Psychiatry, University of Minnesota, Minneapolis, MN, USA; Department of Psychiatry and Human Behavior, University of California Irvine, Irvine, CA, USA; Center for the Neurobiology of Learning and Memory, University of California Irvine, Irvine, CA, USA; Department of Psychology, Georgia State University, Atlanta, GA, USA; Department of Computer Science, Georgia State University, Atlanta, GA, USA

**Keywords:** Brain graph, Functional connectivity, Gaussian graphical model, Joint estimation, Resting-state fMRI, Schizophrenia

## Abstract

Graph-theoretical methods have been widely used to study human brain networks in psychiatric disorders. However, the focus has primarily been on global graphic metrics with little attention to the information contained in paths connecting brain regions. Details of disruption of these paths may be highly informative for understanding disease mechanisms. To detect the absence or addition of multistep paths in the patient group, we provide an algorithm estimating edges that contribute to these paths with reference to the control group. We next examine where pairs of nodes were connected through paths in both groups by using a covariance decomposition method. We apply our method to study resting-state fMRI data in schizophrenia versus controls. Results show several disconnectors in schizophrenia within and between functional domains, particularly within the default mode and cognitive control networks. Additionally, we identify new edges generating additional paths. Moreover, although paths exist in both groups, these paths take unique trajectories and have a significant contribution to the decomposition. The proposed path analysis provides a way to characterize individuals by evaluating changes in paths, rather than just focusing on the pairwise relationships. Our results show promise for identifying path-based metrics in neuroimaging data.

## INTRODUCTION

The human brain is one of the most complex networks, comprising about 86 billion neurons connected by about 150 trillion synapses allowing neurons to communicate and pass chemical or electrical signals to each other (Azevedo et al., 2009; Farahani, Karwowski, & Lighthall, 2019; Fornito, Zalesky, & Bullmore, 2016). Advances in graph-theoretical analysis provide an opportunity to examine and understand this complex network in human cognition and behavior as well as neurological and psychiatric disorders (Farahani, Karwowski, & Lighthall, 2019; Fornito, 2016; Yu et al., 2015, 2018). Graph theory provides a simple and powerful way to model, estimate and simulate the structure and dynamics of brain networks (Falakshahi, Rokham, et al., 2020; Fornito, 2016; Fornito, Zalesky, & Bullmore, 2016; van den Heuvel & Fornito, 2014; van den Heuvel et al., 2017). A brain network or brain graph is composed of a set of nodes (vertices) as brain regions connected by a set of edges (links) as some measure of structural or functional interaction between those brain regions (Farahani, Karwowski, & Lighthall, 2019; Fornito, 2016).

The combination of graph theory and state-of-the-art noninvasive brain imaging techniques has shown promise in the detection of potential biomarkers of mental disorders (Farahani, Karwowski, & Lighthall, 2019; Yu et al. 2018). Brain function can be localized through functional magnetic resonance imaging (fMRI) that assesses the blood oxygenation level–dependent (BOLD) signal from the brain (Hillman, 2014). There is a vast literature on using graph–based methods to analyze brain functional (network) connectivity patterns applying fMRI data (Farahani, Karwowski, & Lighthall, 2019; Fornito, 2016; Karwowski, Vasheghani Farahani, & Lighthall, 2019; Yu et al., 2018). Different graph metrics were used in these studies to characterize the brain graphs, such as clustering coefficient, modularity, characteristic path length, small-world, and assortativity (Farahani, Karwowski, & Lighthall, 2019; Yu et al., 2018). For instance, clustering coefficient, local efficiency, and global efficiency have been shown to be lower in functional brain graphs in people with schizophrenia (SZ) compared to controls (Fu, Iraji, et al., 2021; Fu, Sui, et al., 2021; Liu et al., 2008). Topological alterations in basal ganglia and limbic systems in patients with sleep-related hyper-motor epilepsy have been reported using resting-state fMRI data (Evangelisti et al., 2018). There are numerous reports on graph-based abnormalities in autism spectrum disorder (ASD) using resting-state fMRI data that have shown lower modularity and clustering coefficient in ASD compared to controls (Di Martino et al., 2013; Farahani, Karwowski, & Lighthall, 2019; Keown et al., 2017; Rudie et al., 2013; van den Heuvel et al., 2017).

However, the focus in neuroimaging data has primarily been on edge and nodal changes or global graphic metrics, with little focus on the information contained in paths connecting brain regions. A path from node X to node Y in a graphical model is a sequence of adjacent edges between nodes X and Y. Paths are of great importance in brain graphs, and details of disruption of these paths in patient group graph may be highly informative for understanding disease mechanisms. Importantly, since different edges in the same path may drop out in different individuals, path-based analysis has the potential to capture information that is typically invisible to approaches that focus only on pairwise relationships.

Our main contribution in this study is to show that a comprehensive path (as opposed to individual edge) analysis may help identify putative path-based biomarkers from neuroimaging data. We compare and analyze paths between the brain graphs of control and patient groups. Interactions in graphs often happen in groups of three or more nodes that cannot be explained clearly in terms of pairwise relationships or single links. Paths analyses allow us to analyze the network beyond pairwise interaction. We provide an algorithm to estimate edges that trigger absent paths (disconnection) and additional paths (abnormal integration) in the patient group with reference to the control group graph. In addition, to examine path differences that are common across groups, we propose the use of a previously reported covariance decomposition method (Jones & West, 2005) in which the covariance between two nodes can be decomposed into a sum of weights associated with each of the paths connecting those two nodes.

We conduct our path analyses under the Gaussian graphical model (GGM) framework to have an interpretable model of brain graphs from resting-state fMRI data. GGM is frequently used to explore networks among multiple random variables and, in this study, represents the interaction between brain components. In a GGM, two variables are conditionally independent given all other variables, if and only if their corresponding off-diagonal element in the precision matrix (inverse of the covariance matrix) is zero, and there is no edge between them. Therefore, the precision matrix in GGM summarizes the conditional dependence of network structure (He et al., 2019).

Previous studies for network analysis, for the most part, focused on Pearson correlation–based matrices to measure the strength of association between nodes in a network. GGM is used to study the partial correlation structure of a set of variables and is a better way to model a complex network because of its interpretation with conditional dependence between two random variables after removing the effect of all other controlling variables. Pearson correlation, on the contrary, ignores the effect of all other variables (He et al., 2019). There are many methods for estimating GGM. In this study, we chose to use the joint graphical lasso, which is a technique for jointly estimating multiple graphical models corresponding to distinct but related classes (Danaher, Wang, & Witten, 2014; Guo, Levina, Michailidis, & Zhu, 2011; Shan & Kim, 2018). The joint estimation borrows strength across classes to boost the estimation of multiple graphical models that share certain characteristics while retaining support for class-specific structures. Additionally, estimating control and patient graph separately using the group-specific data does not consider similarity between the group models. Jointly estimating control and patient group graphs improves identifying the differences between groups by considering the assumption of similarity between the models.

Recent brain imaging studies have demonstrated that brain functional connectivity is dynamic over time (Allen et al., 2014; Bhinge, Long, Calhoun, & Adali, 2019; Damaraju et al., 2014; Iraji et al., 2020). However, prior brain graph–based studies focused predominantly on static functional (network) connectivity with an implicit assumption of stationary brain interactions during the scanning period. Although static functional connectivity has disclosed a great deal of knowledge, evaluating brain connectivity changes across time can reveal additional valuable insight into the inherent dynamic connectivity of the human brain. Therefore, in addition to static, we analyze paths on time-varying GGMs for control and patient groups. To the best of our knowledge, no study has provided a dynamic path–based comparison of time-varying brain graphs in group studies to identify possible brain abnormalities.

### Application of the Proposed Method to Schizophrenia

We illustrate the utility of the proposed method on resting-state fMRI data of SZ patients. SZ is commonly distinguished as a disorder of brain connectivity (Fornito, Zalesky, Pantelis, & Bullmore, 2012), although its diagnosis is mostly dependent on qualitative symptom–based measures (Rokham et al., 2020). It is characterized by functional disconnectivity (Friston, 1998) or abnormal integration between distant brain regions (Damaraju et al., 2014; Skudlarski et al., 2010). Prior studies have shown functional connectivity abnormalities in SZ in a broad spectrum of systems that frequently involve prefrontal brain regions (Cole, Anticevic, Repovs, & Barch, 2011; Fornito et al., 2013; Skaatun et al., 2017; van den Heuvel & Fornito, 2014). Still, little is known about how this disconnection or abnormal integration manifests. We estimate time-varying and static graphs of the control and SZ group and apply our proposed method for comprehensive path analysis. A preliminary version of this work has been reported in Falakshahi, Vergara, et al. (2020).

The remainder of the paper is structured as follows: Materials and Methods describes the details of our method, including estimating static and time-varying graphs of control and patient groups and our proposed algorithm for path analysis; Results provides details of our path analysis results on resting-state fMRI data of individuals with SZ and control groups; Discussion reviews our results and implications. We provide concluding remarks in Conclusion.

## MATERIALS AND METHODS

**Figure 1.**
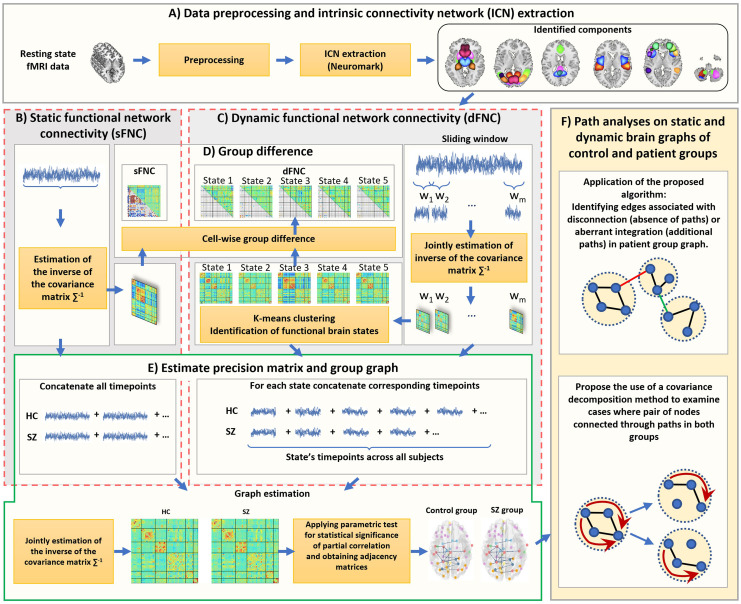
Method outline. (A) data preprocessing and intrinsic connectivity networks (ICNs) extraction using the Neuromark templates, (B) Static functional network connectivity analysis for each subject. (C) Dynamic functional network connectivity estimation using sliding window approach and identification of functional brain states (patterns) via *k*-means clustering algorithm. (D) Evaluating group differences between patient and control groups in dFNC and sFNC by using two-sample *t* test in univariate way. (E) Estimation of inverse of the covariance matrices for control and patient groups by using joint graphical lasso method for static and dynamic states. For the static, the whole time point across all the subjects of each group was concatenated and then joint estimator was applied on them. In dynamic scene, for each state the joint estimator was applied on the aggregate corresponding time point of that state across subjects. The parametric test for statistical significance of the partial correlation was applied for each element of the partial correlation matrix to determine the significant edges. The edge was considered between two brain components only where the corresponding false discovery rate (FDR) corrected *p* value was significant (*p* < 0.05). (F) Path-based differential analysis investigated on control and patient groups graphs.

The methods consist of two parts: identifying static and dynamic graphs from resting-state fMRI data and “path analysis,” including identifying edges that are associated with disconnection or abnormal integration in patient group graph and the novel use of a covariance decomposition method to examine the case where there is at least one path between two specific brain components in both control and patient groups. In Figure 1, we illustrate the main steps, and each section will be explained in detail in the following subsections.

### Data Information, Preprocessing, and Intrinsic Connectivity Network Extraction

#### Data information.

In this work, we analyzed eyes-closed resting-state fMRI data collected from 160 controls and 151 age, gender, and mean framewise displacement (FD)–matched individuals with schizophrenia (SZ) (age: *p* = 0.18; gender: *p* = 0.39; mean FD: *p* = 0.97) by the Function Biomedical Informatics Research Network (fBIRN) (Keator et al., 2016). fBIRN demographics are available in Supporting Information Table S1. Written informed consent was obtained from all subjects. Data were collected using 3T scanners with a repetition time (TR) of 2 s, voxel spacing size of 3.44 × 3.44 × 4 mm, a slice gap of 1 mm, and a total of 162 volumes at seven different sites. Data for six of the seven sites were collected on a 3T Siemens Tim Trio System, and for one other site, a 3T General Electric DiscoveryMR750 scanner was used. Further details on the dataset can be found in Fu, Iraji, et al. (2021) and Fu, Sui, et al. (2021).

#### Data preprocessing.

Data preprocessing was performed using the SPM 12 (https://www.fil.ion.ucl.ac.uk/spm/) toolbox. We performed rigid body motion correction to correct subject head motion, followed by the slice-timing correction to account for timing difference in slice acquisition. The fMRI data were normalized to the EPI template and resampled to 3-mm^3^ isotropic voxels. Data were spatially smoothed using a Gaussian kernel with a 6-mm full width at half maximum. Subjects with head motion < = 3° and < = 3 mm, and with functional data providing near full brain successful normalization (by comparing the individual mask with the group mask) were selected for further analysis. More details on preprocessing and subject selection can be found in Damaraju et al. (2014), Fu, Iraji, et al. (2021), and Fu, Sui, et al. (2021).

#### Intrinsic connectivity network extraction.

We use independent component analysis (ICA) to determine the nodes of a graph (Yu et al., 2017), which decomposes the whole-brain fMRI into independent spatial components known as ICN. Each ICN is composed of a set of voxels that shows a strong coupling of spontaneous fluctuations in the BOLD signal that can be considered as one functional unit (Iraji et al., 2019). Using fully blind ICA as a method for ICN extraction estimation of functional connectivity measures might result in different components identified across data. This inconsistency of identified components may impede finding replication or comparison. To address this and obtain the same set of ICNs for both control and SZ groups, we used the fully automated Neuromark pipeline, which is a spatially constrained ICA informed by reliable network templates (Du et al., 2020). To generate the Neuromark templates and to identify robust and reproducible ICNs, spatial ICA was applied to two large datasets of typical controls (Human Connectome Project [HCP; 823 subjects after the subject selection] and Genomics Superstruct Project [GSP; 1,005 subjects after the subject selection]). Group ICA with model order 100 was performed on each dataset separately to obtain group-level components. Then independent components from HCP and GSP were matched based on the spatial similarity (correlation > = 0.4), and reproducible ICNs were chosen as the network templates. ICNs identification and functional domain labeling were performed by five neuroscience experts. More details of the Neuromark templates can be found in Du et al. (2020). Next, spatial maps and time courses (TCs) for the fBIRN dataset were obtained by the network templates as prior information within a spatially constrained ICA algorithm. For the spatially constrained ICA algorithm, we used multivariate objective optimization independent component analysis with reference (MOO-ICAR), implemented in the GIFT toolbox from TReNDS (https://trendscenter.org/software/gift) (Iraji et al., 2020), as it shows good performance based on our previous studies (Du & Fan, 2013; Du, Allen, et al., 2016).

### Static Functional Network Connectivity

To create a static functional network connectivity (sFNC) matrix for each subject, pairwise correlations were calculated using the entire length of the ICNs time course. We then calculated the mean of sFNC matrices across subjects for control and patient groups. Further analysis on sFNC, including group differences using univariate statistical test, estimating group graphs and path analysis described in sections D and E.

### Dynamic Functional Network Connectivity

Dynamic functional network connectivity (dFNC) investigates the time-varying interactions between brain networks and has been studied extensively (Bhinge, Long, Calhoun, & Adali, 2019; Sakoğlu et al., 2010). dFNC analysis enables us to evaluate how FNCs between ICNs evolve over time. A sliding window approach with square window type was applied to the selected ICNs’ TCs to study the connectivity between selected ICNs in a dynamic manner. ICNs’ TCs were localized by a sliding window size of 20 TR and a window step size of 5 TR to reduce computational demands, which led to 28 windows capturing changes in connectivity over time.

Next, we used the joint graphical lasso (Danaher, Wang, & Witten, 2014) to estimate the inverse of the covariance matrix (precision matrix) for windowed TCs of each subject. We chose to use a joint graphical lasso, which is a technique for jointly estimating multiple graphical models corresponding to distinct but related classes (windows of each subject) (Danaher, Wang, & Witten, 2014; Guo, Levina, Michailidis, & Zhu, 2011; Shan & Kim, 2018). As the simulation study suggested in Danaher, Wang, and Witten (2014), we resample each window data point by using the fast Fourier transform method to augment the sample time from 20 to 400 to robustly estimate covariance matrices and improve the accuracy of estimation. Having the estimated covariance matrix, we then computed the correlation matrix for each window; the detailed formulation is provided as Supporting Information Formula 1.

Recent resting-state studies have shown that fluctuations in the brain networks are not random and reoccur across and within subjects (Damaraju et al., 2014). These dynamic brain states or patterns can be identified by grouping windowed FNC using a *k*-means clustering algorithmv (Allen et al., 2014; Bhinge, Long, Calhoun, & Adali, 2019; Iraji et al., 2020). Therefore, each cluster contains similar functional connectivity patterns. We identified these functional brain states from computed dFNC matrices across all subjects. First, we flatten the upper diagonal part of each FNC matrix to obtain a feature vector. Then, for each subject, the variance across the feature was computed. Similar to what has been done previously (Allen et al., 2014; Damaraju et al., 2014), initial clustering was applied on a subset of data exhibiting maximal deviation from the mean called subject exemplars. The clustering algorithm was applied to these subject exemplars by using the L1 distance function (Manhattan distance) as the distance measure with random initialization of centroid positions and replicated 50 times to increase the chances of escaping local minima. As the *k*-means algorithm is very sensitive to the starting point, we applied subject exemplars to select the peaks (strongest contributors) as initialization. Manhattan distance was chosen as it has been suggested that it might be more effective to estimate similarity measures for high-dimensional data (Allen et al., 2014). The centroids from clustering the subject exemplars were used as initial points to cluster all dFNC windows from all subjects (Allen et al., 2014; Damaraju et al., 2014). The optimal number of clusters was determined using the elbow criterion.

### Evaluating Group Differences in sFNC and dFNC Between Groups

We performed the univariate test in static and dynamic functional (network) connectivity to evaluate differences between the control and patient groups. Regarding sFNC, we applied a two-sample *t* test in a univariate manner on estimated sFNC matrices. To obtain group differences in dFNC, we first computed a subject mean for each state from the subject FNC windows assigned to that state as a representative pattern of connectivity of the subject for that state. Then, to identify significant differences between patient and control groups, we performed *t* tests per ICN pair of the averaged dFNC for each state. We corrected for multiple comparisons by using the FDR with a threshold of 0.05.

### Estimating Static and Time-Varying Graphs for Control and Patient Groups

We used GGM for modeling the brain and identifying the graph structure of control and patient groups. The brain graphs gained from GGM are used for encoding relationships among brain components, wherein nodes represent ICNs and edges that demonstrate a partial correlation between nodes. In a GGM, two nodes are conditionally independent, given all other nodes if and only if their corresponding off-diagonal element in the precision matrix is zero. To estimate the precision matrix, we used joint graphical lasso (Danaher, Wang, & Witten, 2014) on ICNs’ time courses.

To estimate static group graphs of control and patient, we concatenated ICNs’ time courses across the subjects of control and patient groups separately and estimated the precision matrix by performing joint graphical lasso estimation on concatenated ICN time courses. To estimate time-varying group graphs of control and patient groups, we extracted a GGM for each state. Clustering the windowed dFNC results in each windowed dFNC being assigned to a specific cluster. Next, the time points of the windows located in one cluster were concatenated to estimate group graphs for each state. Using the concatenated ICN time courses of corresponding windows in each state, we jointly estimated precision matrices for control and patient groups applying the joint estimation presented in Danaher, Wang, and Witten (2014).

Having the precision matrices for each group in static and dynamic states, we computed the partial correlation matrices. The detailed formulation is provided as Supporting Information Formula 2. To obtain the adjacency matrices, the parametric test for statistical significance of the partial correlation was applied for each element of the partial correlation matrix to determine the significant edges. The edge was considered between two ICNs only where the corresponding FDR corrected *p* value was significant (*p* < 0.05).

### Path-Based Differential Analysis

We compared paths between the graphs of control and patient groups for estimated static and time-varying in four possible cases:

1. Disconnection: In this case, two ICNs are reachable from each other, which means there is at least one path between them in the control group graph, but there is no path between them in the patient group graph.
2. Abnormal integration: In contrast to case 1, case 2 occurs when there is no path between two specific ICNs in the control group graph, but there is at least one path between those ICNs’ pair in the patient group graph.
3. Connection in both control and SZ patients: In this case there is at least one path between specific pairs of ICNs in both control and SZ patient group graphs.
4. Disconnection in both control and SZ patients: The last case is when there is no path between two ICNs in both control and patient group graphs.

We considered case 4 as a trivial case and focused on cases 1–3. For cases 1 and 2, we proposed an algorithm to estimate edges associated with disconnection and abnormal integration by mimicking the structure of paths in the control group graph. For case 3, to make the comparison between control and patient graphs, we applied the covariance decomposition in GGM (Jones & West, 2005). We first describe our proposed method for cases 1 and 2 in detail, and then we discuss the proposed method for case 3.

#### Identifying edges associated with disconnection and abnormal integration.

Our proposed method can be applied to estimated patients and control group graphs obtained through GGM to identify which links are associated with mental illness across the fMRI dataset. There are some edges in the control group graph that contribute to creating path(s) between some nodes (ICNs). However, the absence of those edges in the patient group graph leads to the absence of some paths between some nodes. We labeled those edges as disconnectors that are associated with disconnection in the patient group graph. On the contrary, there are some additional edges in the patient group graph that contribute to creating some new paths that do not exist in the healthy group graph. We labeled those edges as connectors that trigger abnormal integration in the patient group graph.

To compare the paths between nodes of estimated graphs, we utilized the concept of the connected component in graph theory. A connected component of an undirected graph is a subgraph in which there is a path between every pair of nodes in that subgraph (Balakrishnan & Ranganathan, 2012). Hence, when two nodes are in the same connected component, it can be concluded that there is at least one path between them.

A precise and succinct description of our method is summarized in Algorithm 1, which receives two graphs as inputs, Graph1 and Graph2 (Line 1 of pseudo-code). The order of the inputs is important. Disconnectors associated with disconnectivity can be obtained by setting the Graph1 and Graph2 inputs as control and patient group graphs, respectively. On the contrary, abnormal integration can be obtained by swapping the inputs and setting Graph1 and Graph2 as patient and control group graphs, respectively.

**Figure:**
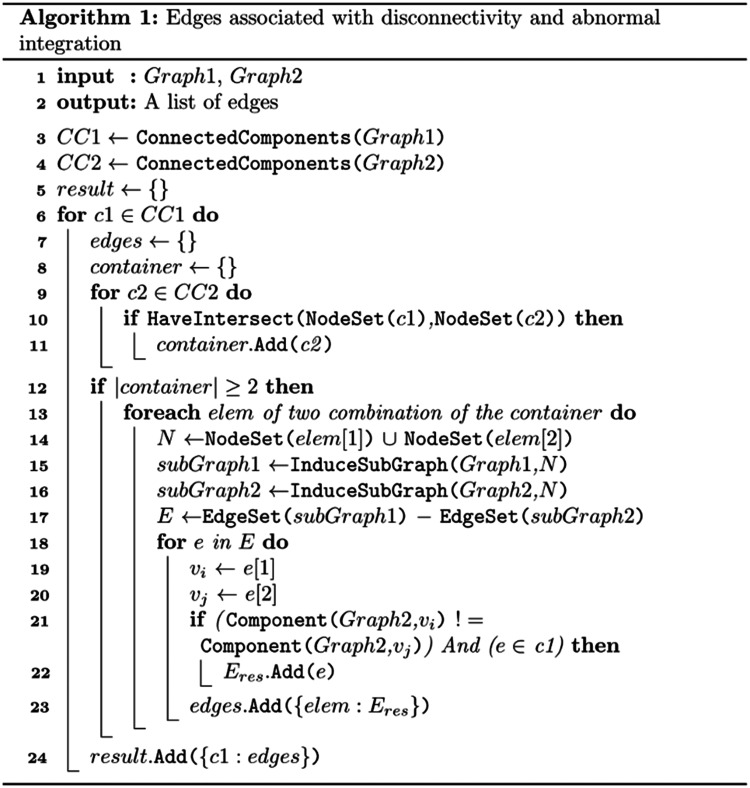

Algorithm 1 first identifies all connected components in Graph1 and Graph2 (lines 3 and 4 of pseudo-code). Then, for each connected component in Graph1, it checks whether the nodes of that connected component are spread into multiple connected components in Graph2 or not. One way to check this is to see if the nodes set of that connected component in Graph1 intersects with more than one connected component nodes sets in Graph2 (lines 6–12 of pseudo-code). The “Container” set (line 11 of pseudo-code) keeps those connected components in Graph2 that intersects with the current connected component of Graph1 (c1). Then, if the cardinality of the Container set becomes greater than or equal to two (line 12 of pseudo-code), it indicates that the nodes of the current connected component of Graph1 (c1) are observed in at least two connected components in Graph2, and it reveals that the separation between the nodes of c1 occurred in Graph2. Next, to identify edges associated with disconnection, all combinations of two connected components in the Container will be investigated (lines 13–23 of pseudo-code). Then for each 2-combination of connected components in the Container set, the union of their nodes is stored in set “N” (line 14 of pseudo-code). Using node-set N, induced subgraphs of Graph1 and Graph2 will be formed (lines 15–16 of pseudo-code). Next, differences between the edge sets of these two subgraphs will be obtained (set “E”) (line 17 of pseudo-code). As not all the edges in set E cause separation, from the set E, those edges will be considered as edges associated with disconnection if their end points belong to two distinct connected components in Graph2. They also should be part of the current connected component in Graph1 (c1) that we are currently analyzing (line 18–23 of pseudo-code). If set E becomes empty, it indicates that the two connected components in Graph2 have become separated indirectly through another connected component.

**Figure 2.**
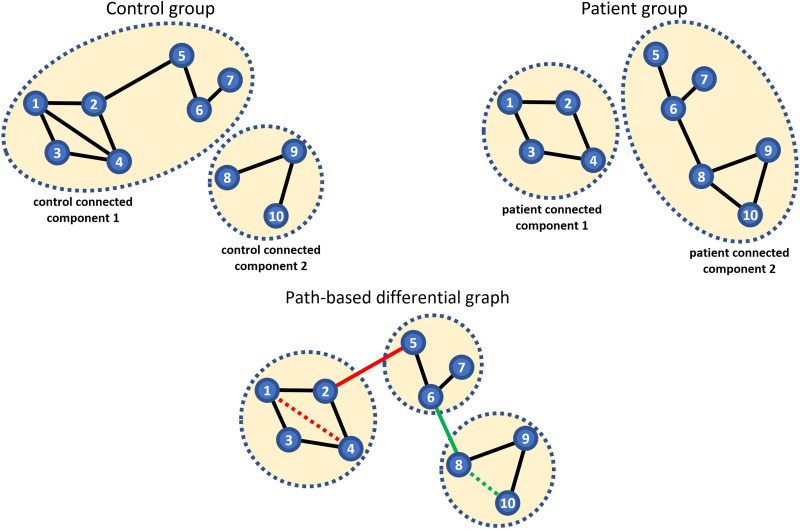
The top-left is an example of the control graph, and the top-right is an example of the patient graph. Bottom row red and green edges are edges that our algorithm returns as disconnector and connector. Note that the edge (8,10) depicted in green dashed line is a new edge in the patient group graph, but it does not contribute to create new path(s) between separated connected components. Similarly, the edge (1, 4) shown with red dashed line is missing edge in patient group graph, but its absence does not lead to disconnectivity between nodes of its connected components, and, indeed, the nodes will still be connected through different paths. Therefore, not all missing edges and new edges in the patient graph are associated with disconnection or abnormal integration.

As an example, to identify disconnectors, consider two fixed graphs of a control group (left) and a patient group (right) in Figure 2. To identify disconnectors, Graph1 and Graph2 are set as control and patient group graph as the input, respectively. As illustrated, there are two connected components in the control and patient group graphs. The nodes set of “control connected component 1” intersects with more than one connected component nodes sets in Graph2:

$$\text{Nodes}\;\text{of}\;\text{control}\;\text{connected}\;\text{component}\;1\cap \text{Nodes}\;\text{of}\;\text{patient}\;\text{connected}\;\text{component}\;1=\left\{1,2,3,4\right\}$$

$$\text{Nodes}\;\text{of}\;\text{control}\;\text{connected}\;\text{component}\;1\cap \text{Nodes}\;\text{of}\;\text{patient}\;\text{connected}\;\text{component}\;2=\left\{5,6,7\right\}$$

As the nodes of connected component 1 spread into multiple connected components in the patient group graph (∣*Container* ∣ ≥ 2), we can conclude that disconnection happened. Then, using the union of the nodes of patient connected components 1 and 2 (set N), subgraphs of control and patient groups will be induced, and the differences of their edges will be obtained with subtraction (set E).

$$N=\left\{1,2,3,4\right\}\cup \left\{5,6,7,8,9,10\right\}=\left\{1,2,3,4,5,6,7,8,9,10\right\}$$

$$E=\text{Edges}\;\text{set}\;\text{of}\;\text{control}\;\text{subGraph}-\text{Edges}\;\text{set}\;\text{of}\;\text{patient}\;\text{subGraph}=\left\{\left(1,4\right),\left(2,5\right)\right\}$$

As not all the edges in set E cause separation, from the set E, those edges will be accepted as edges associated with disconnection if their end points belong to two distinct connected components in Graph2. They also should be the part of the current connected component in Graph1 (c1) that we are currently analyzing (line 18–23 of pseudo-code). In this example, only edge (2, 5) belongs to two different connected components in the patient groups graph. Although the edge (1, 4) is a missing edge, its end points belong to one connected component in the patient group graph, and its absence did not create any separation between nodes of that connected component.

The whole procedure needs to be repeated for all the connected components of Graph1. Therefore, for the second connected component in the control group, the algorithms checks if spreads occurred for control connected component 2:

$$\text{Nodes}\;\text{of}\;\text{control}\;\text{connected}\;\text{component}\;2\cap \text{Nodes}\;\text{of}\;\text{patient}\;\text{connected}\;\text{component}\;1=\left\{\right\}$$

$$\text{Nodes}\;\text{of}\;\text{control}\;\text{connected}\;\text{component}\;2\cap \text{Nodes}\;\text{of}\;\text{patient}\;\text{connected}\;\text{component}\;2=\left\{8,9,10\right\}$$

Because the nodes of connected component 2 intersect with just one connected component in the patient group graph, no disconnection happened for control connected component 2.

Edges associated with abnormal integration (connectors) can be identified similarly, by swapping the order of input variables of the algorithm. In Figure 2, edge (6,8) shown with green is associated with abnormal integration.

#### Applying covariance decomposition approach.

To examine case 3, where two distinct brain components are connected and reachable from each other in both control and patient groups, we first need to introduce a theorem proposed in Jones and West (2005).

**Figure 3.**
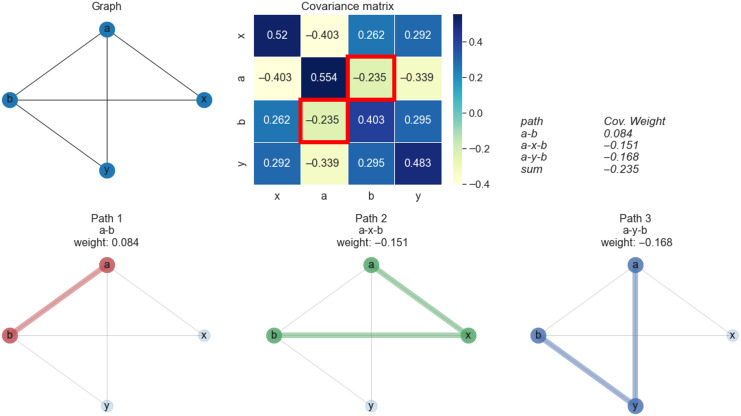
There are three paths between node “a” and “b” as illustrated with three different colors. According to the covariance matrix the covariance between node “a” and “b” is −0.235. Applying the explained formula in Theorem 1 in Jones and West (2005), the covariance between two nodes (in here as an example “a” and “b”) decomposed into a sum of weights associated with each of the paths connecting those two nodes. Sum of weights of a-x-b, a-b, and a-y-b paths will be equal to −0.235.

Theorem 1 (Jones & West, 2005): Let Σ denote the covariance matrix corresponding to a GGM with a set of nodes {1, 2, …, n} with precision matrix Ω, which is the inverse of the covariance matrix Σ^−1^. Also, let *σ*_*xy*_ denote the element of covariance matrix corresponding to the covariance between variables *x* and *y*.

$$\sigma_{xy}=\sum_{P\epsilon {𝒫}_{xy}}{\left(-1\right)}^{t+1}\omega_{p_{1}p_{2}}\omega_{p_{2}p_{3}}\ldots \omega_{p_{t-1}p_{t}}\frac{|\Omega_{\backslash P}|}{|\Omega |}$$ (1)

where 𝒫_*xy*_ is the set of all paths that exist between variables (nodes) *x* and *y*. If we define a path as a sequence of the nodes, then for all path *P* in the set 𝒫_*xy*_, *p*_1_ = *x* and *p*_*t*_ = *y* where *t* refers to the last node in the path. The element of precision matrix between node *p*_i_ and *p*_j_ is denoted by *ω*_*p*_i_*p*_j__ and |Ω_\*P*_| represents the determinant of the matrix Ω_\*P*_ that rows and columns correspond to the nodes of the path *P* excluded. The proof can be found in Jones and West (2005). Figure 3 is an example of applying the covariance decomposition approach.

We applied the covariance decomposition approach for case 3 where paths exist between specific brain components in both control and patient groups to obtain covariance weights for each path between two nodes in both groups. To be able to compare different paths between groups, we used alternative correlation weights, which are constant multiples of the covariance-based weights (Jones & West, 2005). Unlike the covariance-based weights, the correlation-based weights are comparable between paths with different end points.

To identify the differences between control and patient group in case 3, we computed all the paths between each pair of ICNs in both groups and calculated their corresponding correlation weights by using the decomposition method. Lastly, we investigated which paths took unique trajectories in control or patient graphs and studied how their corresponding correlation weights contribute over the whole correlation between the two end points. To this end, for each pair of ICNs that are connected in both control and patient groups, we obtain correlation weights for all simple paths between them via the decomposition method. Next, we identified those brain components that took a distinct path trajectory in comparison with other group and have a significant contribution (more than 50%) to the correlation between paths end points.

## RESULTS

### ICN Extraction

**Figure 4.**
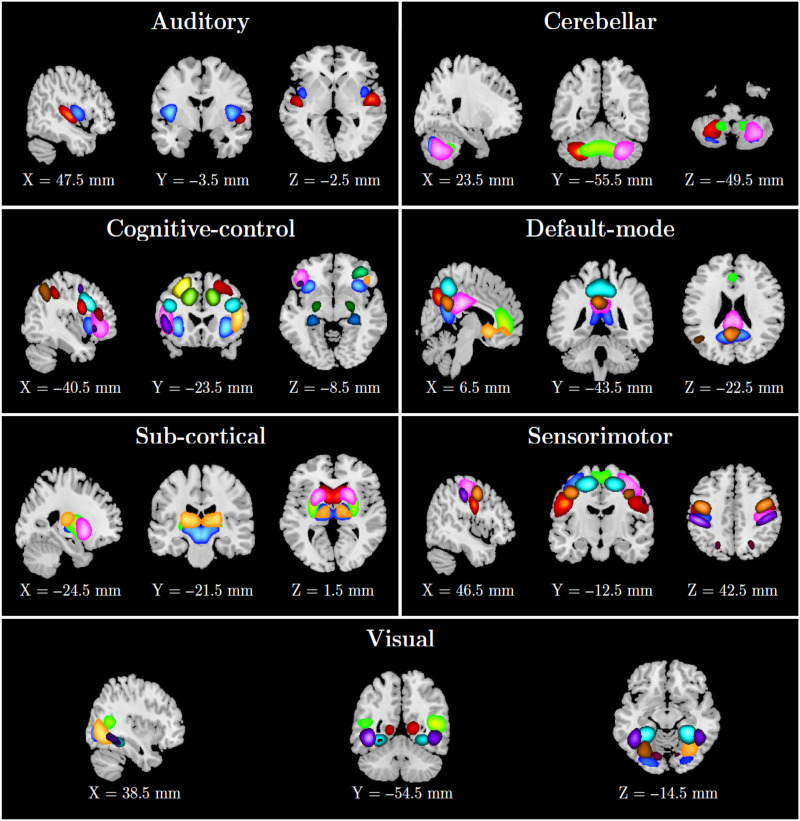
Fifty-three ICNs categorized into seven functional domains. ICN numbers, labels, and domains are presented in Table 1.

We applied the proposed method to resting-state fMRI data collected from control and schizophrenia groups. We selected 53 out of 100 as ICNs for further analysis and categorized them into seven functional domains. The seven labeled functional domains comprise the auditory, subcortical, sensorimotor, visual, cognitive control, cerebellar, and default mode as depicted in Figure 4, and ICN numbers, labels, and domains are presented in Table 1.

**Table 1.** Fifty-three extracted ICNs

**ID**	**Domain**		**ICNs**	**ID**	**Domain**		**ICNs**
1	Subcortical	SC	Caudate	26	Cognitive control	CC	Inferior parietal lobule
2	Subcortical	SC	Subthalamus/hypothalamus	27	Cognitive control	CC	Insula
3	Subcortical	SC	Putamen	28	Cognitive control	CC	Superior medial frontal gyrus
4	Subcortical	SC	Caudate	29	Cognitive control	CC	Inferior frontal gyrus
5	Subcortical	SC	Thalamus	30	Cognitive control	CC	Right inferior frontal gyrus
6	Auditory	AU	Superior temporal gyrus	31	Cognitive control	CC	Middle frontal gyrus
7	Auditory	AU	Middle temporal gyrus	32	Cognitive control	CC	Inferior parietal lobule
8	Sensorimotor	SM	Postcentral gyrus	33	Cognitive control	CC	Left inferior parietal lobe
9	Sensorimotor	SM	Left postcentral gyrus	34	Cognitive control	CC	Supplementary motor area
10	Sensorimotor	SM	Paracentral lobule	35	Cognitive control	CC	Superior frontal gyrus
11	Sensorimotor	SM	Right postcentral gyrus	36	Cognitive control	CC	Middle frontal gyrus
12	Sensorimotor	SM	Superior parietal lobule	37	Cognitive control	CC	Hippocampus
13	Sensorimotor	SM	Paracentral lobule	38	Cognitive control	CC	Left inferior parietal lobe
14	Sensorimotor	SM	Precentral gyrus	39	Cognitive control	CC	Middle cingulate cortex
15	Sensorimotor	SM	Superior parietal lobule	40	Cognitive control	CC	Inferior frontal gyrus
16	Sensorimotor	SM	Postcentral gyrus	41	Cognitive control	CC	Middle frontal gyrus
17	Visual	VI	Calcarine gyrus	42	Cognitive control	CC	Hippocampus
18	Visual	VI	Middle occipital gyrus	43	Default mode	DM	Precuneus
19	Visual	VI	Middle temporal gyrus	44	Default mode	DM	Precuneus
20	Visual	VI	Cuneus	45	Default mode	DM	Anterior cingulate cortex
21	Visual	VI	Right middle occipital gyrus	46	Default mode	DM	Posterior cingulate cortex
22	Visual	VI	Fusiform gyrus	47	Default mode	DM	Anterior cingulate cortex
23	Visual	VI	Inferior occipital gyrus	48	Default mode	DM	Precuneus
24	Visual	VI	Lingual gyrus	49	Default mode	DM	Posterior cingulate cortex
25	Visual	VI	Middle temporal gyrus	50	Cerebellar	CB	Cerebellum
				51	Cerebellar	CB	Cerebellum
				52	Cerebellar	CB	Cerebellum
				53	Cerebellar	CB	Cerebellum

### sFNC Analysis and Group Differences Evaluation Between Control and Patient Groups

**Figure 5.**
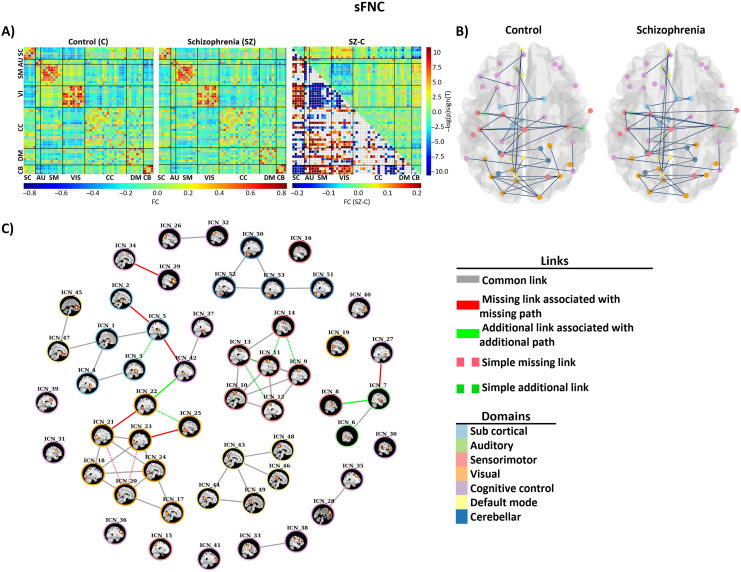
(A) Mean sFNC maps for control and individuals with SZ groups and the result of group differences. The upper triangle is the group difference (SZ-HC) in sFNC, and the lower triangle is the results of multiple comparison test. (B) Estimated static graphs of the control and SZ groups. (C) Results of applying our proposed algorithm on stating graphs of control and patient groups. Disconnectors associated with disconnectivity are shown as solid red edges, and additional edges associated with abnormal integration are shown with solid green edges. The simple missing and simple additional edges are shown with dashed red and green line, respectively.

To identify significant differences between patient and control groups, we performed two-sample *t* tests per ICN pair of sFNC matrices. We corrected for multiple comparisons by using the FDR with a threshold at 0.05. Figure 5A shows the group-specific mean of sFNC matrices and the group differences results. The sFNC analysis results show that the schizophrenia group had weaker functional connectivity between sensory domains (AUD, SM, VIS) in comparison with the control group. Similarly, the connectivity between subcortical and cerebellar domains was lower in the patient group. However, the schizophrenia group showed stronger connectivity between sensory domains and subcortical domain. In addition, the connectivity between sensory domains and cerebellar domain was stronger in schizophrenia group.

### dFNC Analysis and Group Differences Between Control and Patient Groups

Within subject, windowed FNC matrices were computed as described earlier. We computed the variance across the windowed FNC matrices for each subject and selected windows corresponding to local maxima that resulted in 7.2 ± 1.3 (mean ± *SD*) windows per subject with a range of 3 to 11; *k*-means clustering was applied to subjects exemplars, and the centroids from this clustering were used as initial points to cluster all dFNC windows from all subjects. The optimal number of clusters was determined as five by the elbow criterion, which is within a reasonable range of previous dFNC studies (Allen et al., 2014; Fiorenzato et al., 2019; Fu et al., 2018; Tu et al., 2019). The elbow plot can be seen in Supporting Information Figure S1.

**Figure 6.**
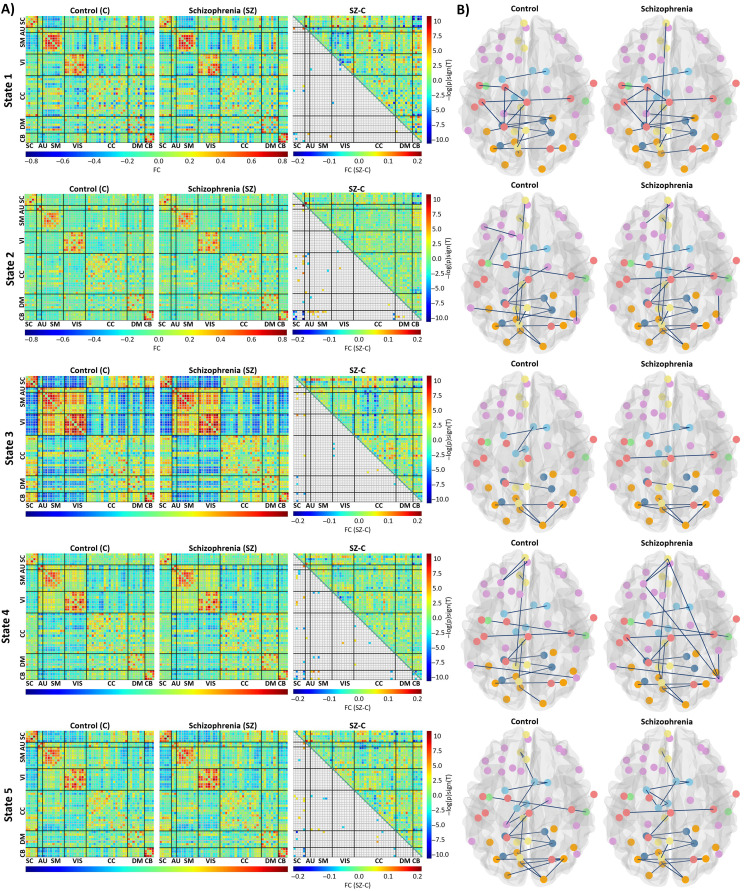
(A) Each state’s results include parts of averaged correlation matrices for control and SZ and group differences. The upper triangle is the differences between averaged correlation matrix of SZ and control (SZ-C), and the lower triangle is the result of multiple comparison test. (B) Parts of estimated graphs of the control and SZ groups.

The average of functional connectivity for each state are shown in Figure 6A. In group difference evaluations, the number of significant FDR corrected *p* values were 16 for State 1, 44 for State 2, 19 for State 3, 37 for State 4, and 30 for State 5. Figure 6A also illustrates the results of the multiple comparison test for the control and SZ groups.

State 2 is the weak-connected state among all states as it shows the weak correlation between all domains. States 1, 4, and 5 have moderate connectivity and State 3 is a strong-connected state. States 5, 4, and 3 have moderate to high functional connectivity among sensory domains (AUD, SM, VIS). The antagonism correlation between SC and sensory domains increases from State 4 to 5 and reaches to its highest value in State 3. Cell-wise differences between each cluster median (FC state) have been illustrated in Supporting Information Figure S2.

Table 2 shows the occupancy measure of a given state as the percentage of windowed FNC that had been labeled with the cluster represented by the given state. About 52% of the windowed dFNCs were clustered as State 2 and State 4, and the occupancy of control group for State 2 and State 4 were 44% and 51%, respectively. In general, the occupancy of different states shows the SZ group were mostly located in weakly connected states (State 1, State 2) in comparison with control, which were observed in strong-connected states (State 3). State 1 was occupied mostly by SZ patients such that 78% of windowed dFNCs of the State 1 belonged to SZ patients, and the occupancy of control group in that state was only 22%. However, 74% of windowed dFNCs in State 3 belonged to the control group. Similarly, the occupancy level of SZ for State 5 was 26% and was lower than the control group. The connectivity between SM and VIS domain in State 3 was stronger than in State 1. In addition, the connectivity between SC and CC, DM, and CB in State 3 was higher than State 1. However, the connectivity between sensory (AUD, SM, VIS) and CB, and between VIS and SB in State 1 was higher than State 3.

**Table 2.** State occupancy

	State 1 (C = 22%, SZ = 78%)	State 2 (C = 44%, SZ = 56%)	State 3 (C = 74%, SZ = 26%)	State 4 (C = 51%, SZ = 49%)	State 5 (C = 74%, SZ = 26%)
State-wise percentage					
All	16%	36%	14%	16%	18%
Control	7%	31%	20%	16%	26%
Schizophrenia	26%	41%	7%	16%	10%

### Estimating Static and Time-Varying Graphs for Control and Patient Groups

We estimated static brain graphs for control and patient groups by using GGM. Figure 5B illustrated the estimated static graphs of control and patient group. The estimated graphs for each state (time-varying graphs) of control and patient groups are illustrated in Figure 6B.

### Identifying Edges Associated with Disconnection and Abnormal Integration in Static and Time-Varying Graphs

We applied our proposed algorithm to identify missing edges that are associated with disconnection and additional edges that are associated with abnormal integration. In static graph analysis, we identified six missing edges and two additional edges that trigger absent paths and additional paths in the SZ group, both within and between functional domains. We found four missing links associated with blocked paths within visual, subcortical, and cognitive control networks and one missing link between cognitive control and auditory (Insula, Middle temporal gyrus) and one between subcortical and cognitive control (Thalamus, Hippocampus). Two additional edges identified between subcortical, auditory, and visual networks. Figure 5C illustrates differential graphs obtained by our proposed method. Table 3 shows details of missing and additional links (edges) associated with SZ in our static analysis.

**Table 3.** The endpoint of links associated with disconnectivity and abnormal integration and their associated functional domain of static analysis

Missing links associated with disconnectivity (***v***_**1**_, ***v***_**2**_)				Additional links associated with abnormal integration (***v***_**1**_, ***v***_**2**_)			
***v***_**1**_		***v***_**2**_		***v***_**1**_		***v***_**2**_	
Domain	ICN	ICN	Domain	Domain	ICN	ICN	Domain
SC	Thalamus	Hippocampus	CC	CC	Hippocampus	Fusiform gyrus	VIS
VIS	Inferior occipital gyrus	Middle temporal gyrus	VIS	AU	Middle temporal gyrus	Postcentral gyrus	VIS
VIS	Right middle occipital gyrus	Fusiform gyrus	VIS				
SC	Subthalamus/hypothalamus	Thalamus	SC				
CC	Supplementary motor area	Inferior frontal gyrus	CC				
CC	Insula	Middle temporal gyrus	AU				

*Note*. DM, default mode network; VIS, visual; CC, cognitive control; SM, sensorimotor; AU, auditory; SC, subcortical; CB, cerebellar.

**Figure 7.**
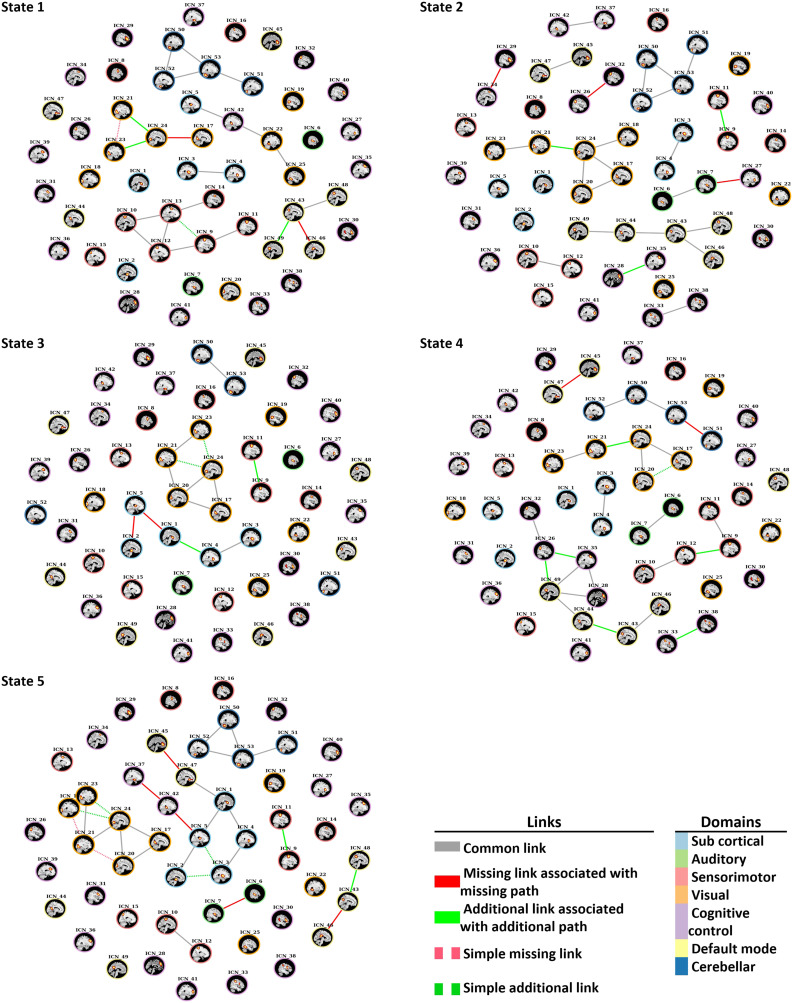
Disconnectors and connectors that were identified in each state. Solid red links trigger disconnection (absence of paths), and solid green links trigger abnormal integration (additional paths) in SZ group with reference to the control group graph.

The disconnectors and connectors that were identified through dynamic approach are shown in Figure 7, and Table 4 shows details of these edges. In State 1, two missing and three additional edges were identified within the default mode and visual domain. The results show that the connection between the lingual gyrus and the calcarine gyrus in the SZ group was disrupted, and the lingual gyrus and the right middle occipital gyrus and the inferior occipital gyrus connected with new additional edges.

**Table 4.** The endpoint of links associated with disconnection and abnormal integration and their associated functional domain of dynamic analysis

State	Missing links associated with disconnectivity (***v***_**1**_, ***v***_**2**_)				Additional links associated with abnormal integration (***v***_**1**_, ***v***_**2**_)			
	***v***_**1**_		***v***_**2**_		***v***_**1**_		***v***_**2**_	
	Domain	ICN	ICN	Domain	Domain	ICN	ICN	Domain
1	DM	Precuneus	Posterior cingulate cortex	DM	DM	Posterior cingulate cortex	Precuneus	DM
	VIS	Calcarine gyrus	Lingual gyrus	VIS	VIS	Right middle occipital gyrus	Lingual gyrus	VIS
					VIS	Inferior occipital gyrus	Lingual gyrus	VIS
2	CC	Supplementary motor area	Inferior frontal gyrus	CC	CC	Superior frontal gyrus	Superior medial frontal gyrus	CC
	CC	Insula	Middle temporal gyrus	AU	VIS	Right middle occipital gyrus	Lingual gyrus	VIS
	CC	Inferior parietal lobule	Inferior parietal lobule	CC	SM	Left postcentral gyrus	Right postcentral gyrus	SM
3	SC	Caudate	Thalamus	SC	SC	Caudate	Caudate	SC
	SC	Subthalamus/hypothalamus	Thalamus	SC	SM	Left postcentral gyrus	Right postcentral gyrus	SM
4	DM	Anterior cingulate cortex	Anterior cingulate cortex	DM	CC	Left inferior parietal lobule	Left inferior parietal lobule	CC
	CB	Cerebellum	Cerebellum	CB	VIS	Right middle occipital gyrus	Lingual gyrus	VIS
					CC	Superior frontal gyrus	Inferior parietal lobule	CC
					DM	Posterior cingulate cortex	Inferior parietal lobule	CC
					DM	Precuneus	Precuneus	DM
					SM	Left postcentral gyrus	Superior parietal lobule	SM
5	DM	Anterior cingulate cortex	Anterior cingulate cortex	DM	SM	Left postcentral gyrus	Right postcentral gyrus	SM
	DM	Precuneus	Posterior cingulate cortex	DM	DM	Precuneus	Precuneus	DM
	CC	Hippocampus	Hippocampus	CC				
	SC	Thalamus	Hippocampus	CC				
	AU	Superior temporal gyrus	Middle temporal gyrus	AU				

*Note*. DM, default mode network; VIS, visual; CC, cognitive control; SM, sensorimotor; AU, auditory; SC, subcortical; CB, cerebellar.

In State 2, two missing links were observed within the cognitive control domain. In addition, one missing link was identified between the insula of the cognitive control domain and the middle temporal gyrus of the auditory domain. In addition, three new connectors were observed in the SZ group within ICNs of the cognitive control, the visual, and the sensorimotor domains.

In State 3, different connections within the subcortical domain were observed in the SZ group. The connection between the caudate and the thalamus and the subthalamus/hypothalamus were disrupted in the SZ group, and new connection between the caudate and the putamen were identified in the SZ group through an additional link. An additional new connection in the SZ group was observed within the sensorimotor domain between the left and the right postcentral gyrus.

In State 4, two disconnectors were identified within the cerebellar and the default mode domains. Five additional links were observed within the cognitive control, the visual, the default mode, and the sensorimotor domains. Also, one additional link was identified between the cognitive and the default mode network domains. The missing links triggered separation within some ICNs of the default mode and the cerebellar domains. However, the additional edges created more connections within the sensorimotor, the visual, and the cognitive control ICNs. Three new additional edges of (superior frontal gyrus, inferior parietal lobule; posterior cingulate cortex, inferior parietal lobule; and precuneus, precuneus) were created for new additional paths between nodes of the cognitive control and the default mode domains in the SZ group.

In State 5, four disruptions within the default mode, the cognitive control, and the auditory domains and one disruption between the cognitive control and the subcortical domains were observed in the SZ group. Three disconnectors were identified that triggered disconnections between nodes of a connected component in the control group that belongs to the default mode, the cognitive control, and the subcortical domains. Two additional integrations also were observed within the sensorimotor and the default mode domains in the SZ group.

### Applying Covariance Decomposition Approach

We also examined the case that paths exist between two specific brain components in both groups by using the covariance decomposition method. In static analysis of control and SZ group graphs, 67 pairs of ICNs were identified between which paths exist in both control and SZ groups. Ten of these pairs took unique path trajectories in at least in one group and have a contribution of more than 50% of the correlation between path end points obtained via the decomposition method. Table 5 summarize the results of those ICN pairs in static analysis. Results of static analysis showed significant differences within the sensory motor, the subcortical, and the visual domains. The SZ groups are connected through higher number of paths that took unique trajectories, with higher correlation weight in comparison with the control group in the sensory motor and the subcortical domains. However, within the visual domain the control group showed a higher number of paths with higher correlation weights.

**Table 5.** Paths that took unique path trajectory and have a significant contribution to the decomposition in static analysis

**ICN pair**				**No. of paths**		**Number of common paths**	**Distinct paths**				**Correlation difference HC-SZ**
***v***_**1**_		***v***_**2**_		**HC**	**SZ**		**HC**		**SZ**		
**Domain**	**ICN**	**ICN**	**Domain**				**No.**	**Weight contribution**	**No.**	**Weight contribution**	
SC	Caudate	Thalamus	SC	1	2	1	0	0.00%	1	74.96%	−0.0012
SM	Left postcentral gyrus	Precentral gyrus	SM	6	33	6	0	0.00%	27	91.16%	−0.0311
SM	Paracentral lobule	Precentral gyrus	SM	6	48	6	0	0.00%	42	56.11%	−0.0045
SM	Right postcentral gyrus	Paracentral lobule	SM	10	27	10	0	0.00%	17	74.04%	−0.0376
SM	Right postcentral gyrus	Precentral gyrus	SM	10	33	10	0	0.00%	23	98.09%	−0.0275
SM	Superior parietal lobule	Paracentral lobule	SM	10	32	10	0	0.00%	22	66.14%	−0.0385
SM	Superior parietal lobule	Precentral gyrus	SM	10	48	10	0	0.00%	38	89.17%	−0.0065
VIS	Cuneus	Right middle occipital gyrus	VIS	21	15	15	6	82.71	0	0.00%	0.103
VIS	Cuneus	Inferior occipital gyrus	VIS	21	15	15	6	81.44	0	0.00%	0.0852
VIS	Fusiform gyrus	Middle temporal gyrus	VIS	22	1	0	22	1	1	100%	−0.0313

**Figure 8.**
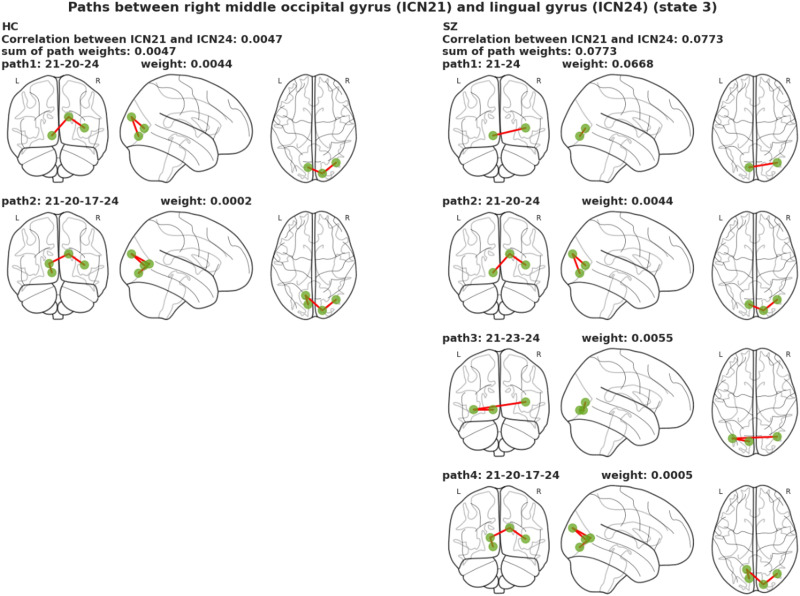
There are two paths between the right middle occipital gyrus and lingual gyrus in the control group in State 3. The number of paths identified between these two ICNs in the SZ group is four. The correlation between path endpoints in control is 0.0046, and the decomposition approach assigned correlation weights of 0.0044 and 0.0002 to each path in control. The correlation between path end points in SZ is 0.0772, and the decomposition approach assigned correlation weights of 0.0668, 0.0044, 0.0055, and 0.0005 to the four paths in SZ. The decomposition approach shows that the two new paths in the patient group contribute 86.51% and 7.1% to the correlation between path end points. A total of 93.61% of correlation weight belongs to these two new paths in the SZ group, and two other paths in SZ that take the same trajectories as controls have a correlation weight of 6.39% of the path end point correlation.

In time-varying graphs of control and SZ analysis, 127 pairs of ICNs were identified between which paths exist in both control and SZ groups. States 3 and 5 revealed that pair 4 and 13 took unique path trajectories at least in one group, respectively, which have a contribution of more than 50% of the correlation between path end points obtained via decomposition. Table 6 summarizes the results of those ICN pairs. Within the visual domain in State 3, the SZ group showed a higher number of paths than the control group in all four cases. Also, distinct paths in terms of the sequence of the nodes in the SZ group account for more than 50% of the total correlation weight between path end points. For example, the number of paths between the calcarine gyrus (ICN17) and the right middle occipital gyrus (ICN 21) was six in the SZ group, while the number of paths between these two ICNs was two in the control group. Two paths in the SZ group were common to the control group, and the other four took some unique path trajectories, and decomposition revealed that those distinct paths in the SZ group have a significant contribution (66.39%). As an example, Figure 8 depicts the result of covariance decomposition analysis between the right middle occipital gyrus and the lingual gyrus in State 3. The correlation between these two ICNs in the control and SZ groups is 0.0046 and 0.0772, and the number of paths between them in the control and SZ groups is two and four, respectively. The decomposition approach shows that two new paths in the patient group contribute 93.61% of correlation weight. The other two paths in SZ group that took the same trajectories as the control group contribute 6.39% of correlation weight.

**Table 6.** Paths between groups that took unique path trajectory and have a significant contribution to the decomposition in dynamic analysis

**State**	**ICN pair**				**No. of paths**		**No. of common paths**	**Distinct paths**				**Correlation difference (C-SZ)**
	***v***_**1**_		***v***_**2**_		**C**	**SZ**		**Control**		**SZ**		
	**Domain**	**ICN**	**ICN**	**Domain**				**No.**	**Weight contribution**	**No.**	**Weight contribution**	
3	VIS	Calcarine gyrus	Right middle occipital gyrus	VIS	2	6	2	0	0%	4	66.39%	−0.0106
3	VIS	Calcarine gyrus	Inferior occipital gyrus	VIS	2	7	2	0	0%	5	92.14%	−0.0071
3	VIS	Right middle occipital gyrus	Lingual gyrus	VIS	2	4	2	0	0%	2	93.61%	−0.0726
3	VIS	Inferior occipital gyrus	Lingual gyrus	VIS	2	4	2	0	0%	2	98.86%	−0.053
5	SC	Subthalamus/hypothalamus	Putamen	SC	1	3	1	0	0%	2	99.82%	−0.0376
5	SC	Subthalamus/hypothalamus	Caudate	SC	1	4	1	0	0%	3	96.98%	−0.0088
5	SC	Putamen	Thalamus	SC	1	3	1	0	0%	2	97.64%	−0.0609
5	SC	Caudate	Thalamus	SC	1	3	1	0	0%	2	70.17%	−0.0152
5	VIS	Calcarine gyrus	Inferior occipital gyrus	VIS	4	4	2	2	31.41%	2	83.8%	−0.0102
5	VIS	Inferior occipital gyrus	Lingual gyrus	VIS	3	2	1	2	9.14%	1	83.8%	−0.0597
5	VIS	Calcarine gyrus	Middle occipital gyrus	VIS	4	2	0	4	100%	2	100%	−0.0091
5	VIS	Middle occipital gyrus	Cuneus	VIS	3	2	0	3	100%	2	100%	−0.0043
5	VIS	Middle occipital gyrus	Right middle occipital gyrus	VIS	1	2	0	1	100%	2	100%	0.047
5	VIS	Middle occipital gyrus	Inferior occipital gyrus	VIS	1	2	0	1	100%	2	100%	0.0024
5	VIS	Middle occipital gyrus	Lingual gyrus	VIS	3	1	0	3	100%	1	100%	−0.0531
5	VIS	Cuneus	Inferior occipital gyrus	VIS	3	4	2	1	85.12%	2	83.8%	−0.0029
5	VIS	Cuneus	Right middle occipital gyrus	VIS	3	4	2	1	85.12%	2	6.77%	0.0292

Similar scenarios were observed within the subcortical and the visual domain in State 5. Four cases show that the SZ group has more paths between components of putamen, caudate, and thalamus, which also involve a significant proportion of total correlation between those end points. In addition, two cases that involve the inferior occipital gyrus as one of the path end points show that the distinct paths in the SZ group had a significant effect on the total correlation between the path endpoints.

Five cases in State 5 show the paths from the middle occipital gyrus to other visual domain components have totally different trajectories in control and SZ groups and do not share any common paths between control and SZ groups. Moreover, the path analysis between the cuneus and the right middle occipital gyrus shows that the SZ group has more paths than the control group; however, the unique path of control significantly contributes to the total correlation weights between end points. Similar results were observed between the cuneus and the inferior occipital gyrus, but the distinct paths of both control and SZ groups had a significant contribution to the decomposition.

## DISCUSSION

In this study, we estimated static and dynamic brain graphs from resting-state fMRI data. We applied a data-driven approach for determining the nodes and edges of the graphs. Spatial brain components evaluated by group ICA was used to define data-driven nodes, and for estimating graphs edges, we employed statistical tests and mitigated the bias of relying on ad hoc thresholds for correlation values. After estimation of a partial correlation matrix, we obtain the corresponding *p* values, and we only considered an edge between two nodes if it passes a significance level (0.05) after multiple comparison correction (FDR). This contribution reduces the need for subjective choices as in other widely used approaches (Karwowski, Vasheghani Farahani, & Lighthall, 2019).

Existing methods for identifying differences between control and SZ patient groups in functional brain imaging literature are limited to correlation networks and pairwise functional connectivity comparison. Most studies focus only on pairwise relationships or “single links.” The path in such networks cannot reveal meaningful interpretation in comparison with the GGM, in which the conditional dependency plays an important role in determining connection and paths between nodes.

We analyzed paths on both static and dynamic manner of control and patient group graphs. We provide an algorithm to estimate edges associated with disconnection (absence of paths) or aberrant integration (additional paths) in dynamic patient group graphs by mimicking the structure of control group graphs. We used the concept of the connected component in graph theory to examine the presence or absence of a path between two nodes of a graph, as there is at least one path between every pair of nodes in a connected component. This results in time complexity reduction of path analysis in comparison with checking the absence or presence of paths for every pair of nodes of a graph. In the first part of the algorithm, we identified connected components in graphs using the NetworkX python package (Hagberg, Swart, & Chult, 2008). Next, through multiple steps in the proposed algorithm, we checked if the nodes of each connected component separated or joined to a new connected component in the second graph. Then, if any separation or abnormal integration occurred, the edges associated with those cases would be returned as an output of the algorithm. This algorithm can be applied to either static or dynamic graphs.

In addition, we studied the cases where two nodes (ICNs) connected in both control and patient groups. We investigated which paths take unique trajectories and computed their corresponding correlation weights by using the covariance decomposition method in a GGM. We applied our method to both static and dynamic time-varying graphs to analyze changes in paths over time.

We illustrated the utility of our proposed method on resting-state fMRI data of SZ patients. Previous studies have been demonstrated that SZ is associated with a disruption of the connections present in the healthy brain (Hunt, Kopell, Traub, & Whittington, 2017; Pearlson, 1997; van den Heuvel & Fornito, 2014; van den Heuvel, Mandl, Stam, Kahn, & Pol, 2010). Since SZ is a psychiatric disorder that can be distinguished by functional disconnectivity or abnormal integration between distant brain components (Damaraju et al., 2014; Friston, 1998; Skudlarski et al., 2010), we analyzed additional and blocked paths in estimated SZ time-varying graphs. However, the proposed method can be applied to any data related to other conditions that can be distinguished by disconnectivity or abnormal integration. Using our proposed algorithm, we found several disconnectors and connectors associated with disconnectivity and abnormal integration (see Table 3 and Table 4).

Our findings show 14 disconnectors and 16 connectors, both within and between functional domains, across all five states in time-varying brain graphs analysis. Static approach reveals six disconnectors and two connectors. Table 7 summarizes the number of identified connector and disconnector across all states in dynamic and static analysis. In static analysis, the disconnection observed within the nodes of the visual, the subcortical, and the cognitive control domains and between nodes of the subcortical, the cognitive control, and the auditory domains. In addition, two abnormal integrations observed between the cognitive control, the visual, and the auditory domains. In dynamic graph analysis within the default mode network, we found four missing links associated with blocked paths in States 1, 4, and 5. Several of the observed disconnectors showed agreement between static and dynamic approach. Four of six disconnections in static analysis, which were observed within and between the subcortical, the cognitive control, and the auditory domain, were also identified in different states of dynamic analysis. In dynamic approach, the disruption identified between the thalamus (SC) and the hippocampus (CC) in State 5; between the insula (CC) and the middle temporal gyrus (AU), and between the supplementary motor area (CC) and the inferior frontal gyrus (CC) in State 2; and between the subthalamus/hypothalamus (SC) and the thalamus (SC) in State 3 were observed in static analysis as well. However, results dynamic approach can capture more detailed information regarding the disconnection and abnormal integration that would be missed in static analysis. Therefore, path analysis on time-varying graphs extends findings from static analysis and provides additional results that are complementary to, and extend, a static analysis.

**Table 7.** Number of identified links associated with disconnection and abnormal integration in static and dynamic analysis

	Number of missing links associated with missing paths	Number of additional links associated with additional paths
Static analysis	6	2
State 1	2	3
State 2	3	3
State 3	2	2
State 4	2	6
State 5	5	2

The default mode network describes a distributed large-scale functional brain network that consists of brain components such as the medial prefrontal cortex, posterior cingulate cortex, and precuneus (Fox, 2007) that is more active during rest or internal cognitive processing compared to external goal-directed cognitive tasks (Raichle et al., 2001). Previous fMRI studies in SZ have consistently reported the disruption and aberrant functioning of the default mode network (Du, Pearlson, et al., 2016; Fornito, Zalesky, Pantelis, & Bullmore, 2012; Mars et al., 2012; Öngür et al., 2010; Pankow et al., 2015). Interestingly, our approach identified three new links associated with additional paths within the default mode network and one new link between the default mode network and cognitive control in precisely the same states as disconnectors were found in dynamic approach, which may be related to a compensatory response in the SZ group that warrants future study.

In addition, disruption in the subcortical domain is consistent with and significantly extends recent reports (Damaraju et al., 2014; Woodward, Karbasforoushan, & Heckers, 2012). In more detail, disconnection between the thalamus and the hippocampus, which were identified by our method in both dynamic and static analysis, has been reported previously in Woodward, Karbasforoushan, and Heckers (2012).

Furthermore, the most significant alteration was observed in cognitive control. Cognitive dysfunction in schizophrenia (McTeague et al., 2017) supports the cognitive control disconnections identified by our method, which were observed both in static and dynamic analysis. In dynamic approach, there were five links associated with blocked paths, of which four were within cognitive control, and one was between the cognitive control and auditory network. Also, three new links were observed associated with additional paths within cognitive control, and one new link was between the default mode network and cognitive control networks. Cognitive control is a cognitive and neural mechanism that contributes to the ability to adapt information processing and regulating behavior according to one’s current goals (Miller, 2000), and according to prior studies, cognitive impairments are the main feature of SZ (Cho, Konecky, & Carter, 2006; Ray, Gold, Silverstein, Barch, & Carter, 2017). Our results thus are consistent with and extend findings from prior studies.

To examine case 3, where two distinct brain components are connected and reachable from each other in both control and patient groups, we applied the covariance decomposition method. The dynamic approach results showed that (only) States 3 and 5 have significant group differences (Jones & West, 2005). Although the paths existed between pairs of brain components, particularly within the subcortical and visual domain in both groups, these paths take unique trajectories, and the decomposition method revealed that those distinct paths have significant weight. The results of both static and dynamic analysis suggest that despite the connection between the nodes in both groups in subcortical domain, schizophrenia have a different type of connection, in terms of unique paths trajectories, and the decomposition method showed that those distinct paths have significant weight between the thalamus and the caudate. In addition, dynamic analysis provided more additional differences, which were not revealed in static analysis in the subcortical domain.

Our work shows that differences between control and patient groups are likely the result of “multilink” disruptions in the paths. The results highlight the importance of studying longer path links, especially in the context of complex mental illness such as schizophrenia. Hence, in comparison with the existing methods, more detailed interpretation regarding the differences between control and SZ patients can be obtained using our proposed method. Future work should focus on expanding the approach and replicating our results in additional datasets.

In this work, we analyzed undirected graphs based on partial correlation. Although the graph-theoretical approach can be used to either assess functional or effective connectivity patterns, most human brain graph investigations have applied undirected networks because of inference constraints of directed graphs (Farahani, Karwowski, & Lighthall, 2019; Karwowski, Vasheghani Farahani, & Lighthall, 2019; Liao, Vasilakos, & He, 2017). Future work could utilize directed network analysis that might provide more information on human brain connectivity.

## CONCLUSION

In sum, we provide an approach using GGM to estimate static and time-varying graphs in control and SZ patient groups on resting-state fMRI data. We comprehensively analyze paths and propose an algorithm to estimate links associated with disconnectivity and abnormal integration in the patient group graphs. Also, in cases in which paths exist between two nodes in two groups, we suggest using a covariance decomposition method. We apply our method to study resting-state fMRI data in SZ versus controls, as SZ is a disorder characterized by disconnectivity. We provided detailed information about how reduced integration or abnormal integration manifests in the SZ group. Several missing links associated with disconnectivity were identified in the SZ group, both within and between functional domains, particularly within the default mode network and cognitive control domains. Also, our proposed algorithm identified additional new edges within these domains associated with abnormal integration, which may be related to a compensatory brain response in the schizophrenia group. In analyzing cases in which paths exist between two specific brain components in both groups, these paths in some cases took unique trajectories, and the decomposition method showed that those distinct paths have significant weight. The proposed path analysis provides a way to characterize individuals by evaluating changes in paths, rather than just focusing on pairwise relationships. As the edges within a path might be distinct in different individuals, a path-based approach can capture important information that is ignored by approaches that focus on pairwise relationships. Our first dynamic path analysis found path-based differences between individuals with schizophrenia and healthy controls, indicating its promise for identifying path-based abnormalities in dynamic connectivity in the healthy and disordered brains. Path analysis on time-varying graphs extends findings from static graphs and provides additional results that are complementary to, and extend, a static analysis.

## ACKNOWLEDGMENTS

Data collection was supported by the National Center for Research Resources at the National Institutes of Health (grant numbers: NIH 1 U24 RR021992, NIH 1 U24 RR025736-01).

## SUPPORTING INFORMATION

Supporting information for this article is available at https://doi.org/10.1162/netn_a_00247.

## AUTHOR CONTRIBUTIONS

Haleh Falakshahi: Formal analysis; Methodology; Software; Validation; Visualization; Writing – original draft; Writing – review & editing. Hooman Rokham: Conceptualization; Writing – review & editing. Zening Fu: Data curation; Writing – review & editing. Armin Iraji: Writing – review & editing. Daniel H. Mathalon: Resources; Writing – review & editing. Judith M. Ford: Resources; Writing – review & editing. Bryon A. Mueller: Resources; Writing – review & editing. Adrian Preda: Resources; Writing – review & editing. Theo G. M. van Erp: Resources; Writing – review & editing. Jessica A. Turner: Writing – review & editing. Sergey Plis: Writing – review & editing. Vince D. Calhoun: Conceptualization; Funding acquisition; Investigation; Supervision; Validation; Writing – review & editing.

## FUNDING INFORMATION

Vince D. Calhoun, National Institutes of Health (https://dx.doi.org/10.13039/100000002), Award ID: R01MH118695. Vince D. Calhoun, National Science Foundation (https://dx.doi.org/10.13039/100000001), Award ID: 2112455.

## TECHNICAL TERMS

Brain functional (network) connectivity: Temporal statistical relationships between brain regions or networks.
Gaussian graphical model (GGM): An undirected graph of partial correlation that illustrates the conditional independences of multiple random variables. Edges are regarded as the conditional dependencies between the nodes.
Static functional (network) connectivity: A statistical relationship between brain regions or networks using the entire length of the data.
Intrinsic connectivity network (ICN): An estimation of a set of voxels obtained using ICA with coherent functional activity over time that can be considered as one functional unit.
Independent component analysis (ICA): A statistical technique that is used for estimating maximally independent components.
Brain states: Brain connectivity patterns (states) that reoccur over time and subjects and can be identified by applying *k*-means clustering algorithm.
Dynamic functional (network) connectivity: A statistical relationship between brain regions or networks using a portion of the data to study time-resolved changes in connectivity.
Connected component: A set of nodes in a graph in which there is at least one path between them. That is, it is possible to get from one node to another within a connected component.
