# Supplementary material for: Path analysis: A method to estimate altered pathways in time-varying graphs of neuroimaging data
Source: Netw Neurosci. 2022 Jul 1;6(3):634–64. doi: 10.1162/netn_a_00247 (PMC9531579; doi:10.1162/netn_a_00247)
Supplement: Supplementary file 1 [file netn-06-634-s001.pdf]

Falakshahi, H., Rokham, H., Fu, Z., Iraji, A., Mathalon, D. H., Ford, J. M., Mueller, B. A., Preda, A., van Erp, T. G. M., Turner, J. A., Plis, S., Calhoun, V. D. & Fornito, A . (2022). Supporting information for "Path Analysis: A Method to Estimate Altered Pathways in Time-varying Graphs of Neuroimaging Data." *Network Neuroscience*, *6*(3), 634–664. https://doi.org/10.1162/netn_a_00247

Supplemental Material

Table S1. Demographics of fBIRN cohort.

	Control	Schizophrenia
Number	160	151
Age	38.8±11.6	37.0±10.9
Gender	45F/115M	36F/115M

F, female; M, male

Formula 1:

If the $\boldsymbol{X} = (X_1, \dots, X_n)$ denote an n-dimensional random vector drawn from a multivariate Gaussian distribution with mean μ and covariance matrix Σ and the precision matrix Ω, which is the inverse of the covariance matrix Σ^{-1}, then the correlation between variable X_i and X_j can be obtained from the covariance matrix of the standardized random variables $X_i/\sigma(X_i)$

$$\rho_{ij} = \frac{Cov(X_i, X_j)}{\sigma_i \sigma_j} \quad (1)$$

where ρ_{ij} denote the element of the correlation matrix and $Cov(X_i, X_j)$ denote the element of the covariance matrix corresponding to the covariance between variables X_i and X_j and σ_i is the standard deviation of X_i.

Formula 2:

The partial correlation coefficient between variables X_i and X_j which shows the measures of the conditional correlation of the variable X_i and X_j given the values of the other variables can be obtain by normalizing the off-diagonal entries of the precision matrix.

$$p_{ij}|\boldsymbol{V}_{\setminus\{i,j\}} = -\frac{\omega_{\mathrm{ij}}}{\sqrt{\omega_{\mathrm{ii}}\omega_{\mathrm{jj}}}} \quad (2)$$

Where, ω_{ij} is the element of precision matrix between node X_{i} and X_{j} is denoted by ω_{ij} and $\boldsymbol{V}$ is the set of indices of all variables (Lauritzen, 1996; Malioutov, Johnson, & Willsky, 2006).

Elbow criterion:

The optimal number of clusters was determined as five by the elbow criterion (Fig.S1), which within a reasonable range of previous dFNC studies (Allen, et al., 2014; Tu, et al., 2019; Fu, et al., 2018; Fiorenzato, et al., 2019).

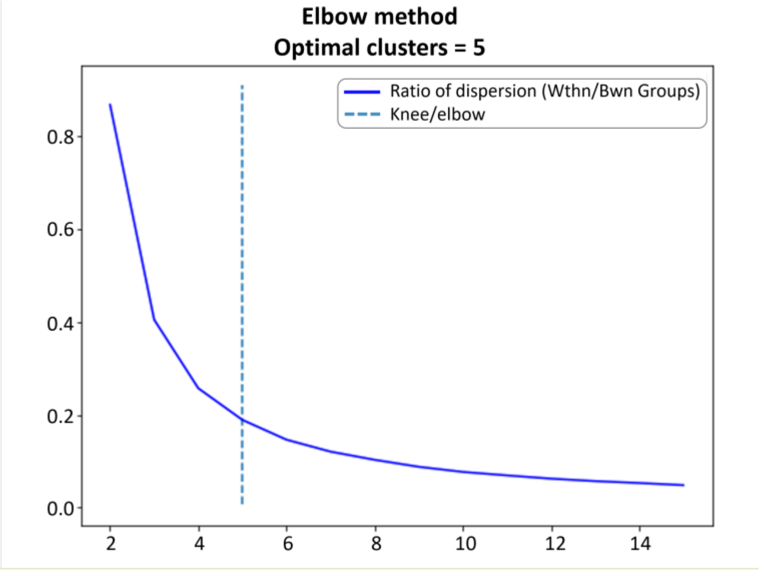

Fig.S1 Elbow method of the k from 2 to 15.

Cell wise difference between each cluster median (FC state)

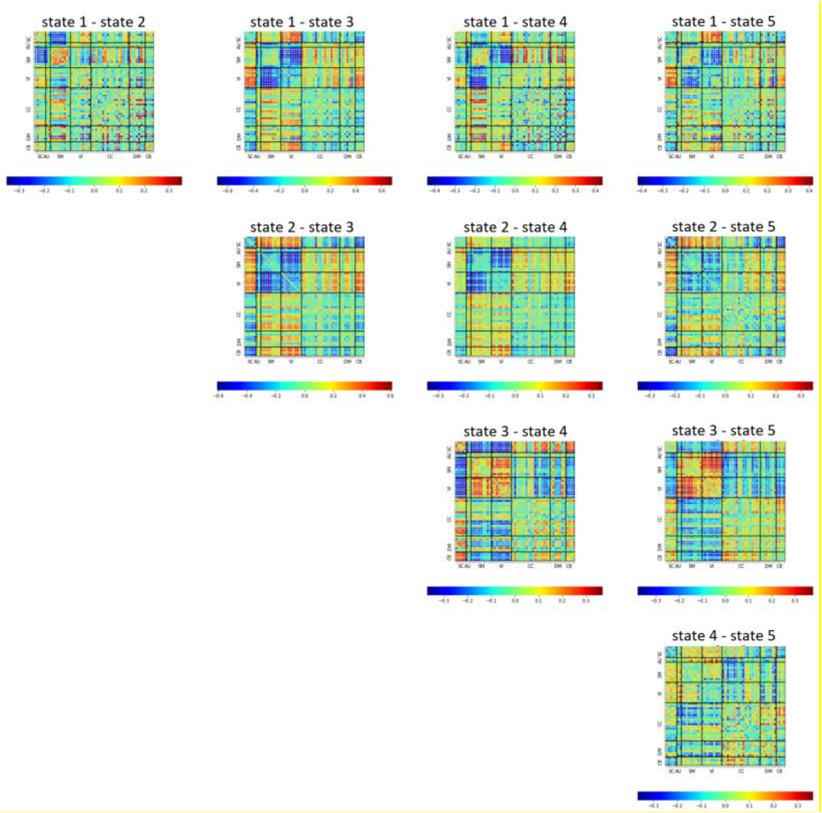

Fig.S2 Functional connectivity (FC) cell-wise difference between each pair of states

References

Allen, E. A., Damaraju, E., Plis, S. M., Erhardt, E. B., Eichele, T., & Calhoun, V. D. (2014). Tracking whole-brain connectivity dynamics in the resting state. *Cerebral cortex, 24*(3), 663-676.

Fiorenzato, E., Strafella, A. P., Kim, J., Schifano, R., Weis, L., Antonini, A., & Biundo, R. (2019). Dynamic functional connectivity changes associated with dementia in Parkinson's disease. *Brain, 142*(9), 2360-2872.

Fu, Z., Tu, Y., Di, X., Du, Y., Pearlson, G. D., Turner, J. A., . . . Calhoun, V. D. (2018). Characterizing dynamic amplitude of low-frequency fluctuation and its relationship with dynamic functional connectivity: an application to schizophrenia. *Neuroimage, 180*, 619-631.

Lauritzen, S. L. (1996). *Graphical models.* Clarendon Press.

Malioutov, D. M., Johnson, J. K., & Willsky, A. S. (2006). Walk-sums and belief propagation in Gaussian graphical models. *The Journal of Machine Learning Research, 7*, 2031-2064.

Tu, Y., Fu, Z., Zeng, F., Maleki, N., Lan, L., Li, Z., . . . others. (2019). Abnormal thalamocortical network dynamics in migraine. Neurology, 92(23), e2706-e2716.
